# Incorporating health workers’ perspectives into a WHO guideline on personal protective equipment developed during an Ebola virus disease outbreak

**DOI:** 10.12688/f1000research.12922.2

**Published:** 2018-03-09

**Authors:** Saskia Den Boon, Constanza Vallenas, Mauricio Ferri, Susan L. Norris

**Affiliations:** 1 World Health Organization, Geneva 27, 1211, Switzerland

**Keywords:** Guidelines, World Health Organization, Values, Preferences, Hemorrhagic Fever, Ebolavirus, Personal Protective Equipment

## Abstract

**Background:** Ebola virus disease (EVD) health facility transmission can result in infection and death of health workers. The World Health Organization (WHO) supports countries in preparing for and responding to public health emergencies, which often require developing new guidance in short timelines with scarce evidence. The objective of this study was to understand frontline physicians’ and nurses’ perspectives about personal protective equipment (PPE) use during the 2014-2016 EVD outbreak in West Africa and to incorporate these findings into the development process of a WHO rapid advice guideline.

**Methods *: ***We surveyed frontline physicians and nurses deployed to West Africa between March and September of 2014.

**Results**: We developed the protocol, obtained ethics approval, delivered the survey, analysed the data and presented the findings as part of the evidence-to-decision tables at the expert panel meeting where the recommendations were formulated within eight weeks. Forty-four physicians and nurses responded to the survey. They generally felt at low or extremely low risk of virus transmission with all types of PPE used. Eye protection reduced the ability to provide care, mainly due to impaired visibility because of fogging. Heat and dehydration were a major issue for 76% of the participants using goggles and for 64% using a hood. Both gowns and coveralls were associated with significant heat stress and dehydration. Most participants (59%) were very confident that they were using PPE correctly.

**Conclusion *: ***Our study demonstrated that it was possible to incorporate primary data on end-users’ preferences into a rapid advice guideline for a public health emergency in difficult field conditions. Health workers perceived a balance between transmission protection and ability to care for patients effectively while wearing PPE. These findings were used by the guideline development expert panel to formulate WHO recommendations on PPE for frontline providers caring for EVD patients in outbreak conditions.

## Introduction

Health facility transmission is a hallmark of early Ebola virus disease (EVD) outbreaks and usually results in infection and death of health workers particularly before the identification of Ebola virus as responsible for the clinical presentation of one or a cluster of patients ^1– 3^. Contributing factors include non-specific clinical presentation, lack of local advanced diagnostic capabilities and suboptimal infection prevention and control (IPC) practices, amplified by poor surveillance in struggling health systems. The epidemiological pattern of the 2014–2016 EVD outbreak in West Africa revealed a similar story, but this time with an unprecedented scale and geographic spread, resulting in a record number of affected health workers, with 881 cases and 513 deaths by late 2015 ^4^. Health workers are more likely than non-health workers to be infected: depending on the profession, the risk can be 21 to 32 times higher ^5^.

The correct use of personal protective equipment (PPE) as part of comprehensive IPC measures contributes to the prevention of EVD transmission in healthcare settings by providing a protective barrier from contaminated fluids. However, the characteristics of the material and the configuration of the equipment may lead to health worker discomfort, overheating, and concerns about dexterity and safety to perform clinical tasks when PPE is used in the typical conditions of high heat and humidity present in West African EVD Treatment Centers ^6, 7^. As the United Nations’ international health agency, the World Health Organization (WHO) has the mandate to support Member States in preparing for and responding to a wide range of public health emergencies that often require that new technical guidance is developed in short timelines with scarce evidence base. Following an urgent request from affected Member States, WHO started the production of a PPE guideline for EVD outbreaks in July 2014, shortly before declaring the EVD outbreak in West Africa a Public Health Emergency of International Concern.

A rapid review of the efficacy and comparative effectiveness of various components of PPE was commissioned in preparation for an expert panel meeting to develop recommendations on optimal PPE for health workers in Ebola treatment units (ETUs) in outbreak settings. It became clear very early in the process that high quality efficacy and comparative effectiveness studies addressing the use of specific PPE items for EVD in outbreak settings were lacking ^8^. In addition to the paucity of data, it was critically important to gather and include the perspectives of health workers who had “real-life” experience in ETUs in West Africa. Early reports of the local conditions indicated that broader clinical questions than PPE performance as a transmission barrier were as important: usability, comfort, dexterity and impact on communication with patients, for example. The underlying principle was that evidence from efficacy and comparative effectiveness studies was necessary but insufficient for contextualization and adequate decision-making. This approach highlights the importance of understanding the way individuals exercise judgement (values and preferences) when selecting options with potential benefits, harms, and inconveniences in real life and is current best-practice in WHO standard guidelines ^9^. Values and preferences are often informed mainly by the opinion of guideline expert panel members, however such proxies for persons affected by the recommendations in a guideline are often inadequate or even inaccurate. Thus, in the early stages of the 2014–2016 EVD outbreak in West Africa, in the context of time constraints and the absence of published data, it was crucial to incorporate the values and preferences of health workers into the guideline development process.

The purpose of this study was to support the development process of a WHO rapid advice guideline on PPE for EVD care in outbreaks. The specific objectives were to understand and describe frontline physician and nurses’ perspectives about PPE use, while providing direct care for EVD patients in the unprecedented conditions of the 2014–2016 EVD outbreak in West Africa and to incorporate these findings into the rapid advice guideline development process.

## Methods

### Approach

In September 2014, we electronically surveyed international frontline physicians and nurses who participated in foreign medical teams deployed to the affected countries in early stages of the EVD outbreak. The pragmatic approach was necessary given that this survey was developed and delivered at the height of outbreak and that WHO had very limited time available in which to produce guidance.

### Survey

The online, 23-item survey was developed specifically for this study ( Supplementary File 1). The first section consisted of multiple-choice questions examining participant demographic characteristics, role, and experience with PPE in West Africa. The next section addressed health worker exposure to the following specific components of PPE: eye protection (goggles/face shields), nose and mouth protection (medical mask/particulate respirator), gloves (single/double gloves), body covering (gowns/coveralls), foot wear (boots/closed shoes), and head covering (hair cover/hoods). In subsequent sections, we used a four or five-point Likert-scale to examine participants’ perceptions about the impact of each PPE item on the following domains: safety, communication, ability to provide patient care, personal wellbeing (heat and dehydration), and comfort. In addition, for each of the items, participants could provide free-text comments on open-ended questions to describe any difficulties or to provide suggestions on how PPE could be improved. The final section explored specific training needs and confidence in PPE. The last question asked participants to compare two sets of PPE available in West Africa shown side-by-side in a picture: one was composed of lighter items and the other had more robust components.

Five experts reviewed the study protocol and questionnaire during the development phase. Subsequently, three clinicians with experience in the EVD outbreak in West Africa similar to that of the sampling frame field-tested the survey for consistency, readability, completeness, and question sequencing. The final version of the online survey incorporated all relevant feedback and comments. We obtained expedited approval of the study protocol and survey from the WHO Ethics Review Committee (RPC690).

We contacted potential participants via email. The first email explained the objectives, expected time commitment, and provided a link to the informed consent form and online survey on Survey Monkey ^®^. Participation was voluntary and implied informed consent. A follow-up email in 5 days reminded potential participants of the deadline (10 days after launching). Participants could withdraw from the study at any time without providing any justification.

### Participants

The study population consisted of international frontline physicians and nurses with direct field experience caring for EVD patients in West Africa. Our sampling frame targeted international physicians and nurses deployed by WHO and Médecins Sans Frontières (MSF) to West Africa between March and September 2014. We used maximum variation purposeful sampling, a non-probability sampling strategy, to capture a wide range of health worker perspectives and experiences in two organizations and four different countries affected by the EVD outbreak. Health workers were reached through a contact individual in each organization (MSF and WHO) who directly emailed potential participants. Physicians and nurses from the affected countries and from other international organizations were not included for pragmatic reasons given the extreme time constraints and infeasibility of obtaining additional organizational approvals in the available timeline. An initial communication error led to the participation of other groups of health workers that did not have frontline clinical experience. The perspectives of these workers were considered for WHO quality improvement efforts, but were excluded from this analysis as these groups were not part of the approved sampling frame for this study.

### Data analyses

Participants could indicate their experience with more than one item for each PPE component (e.g., both goggles and face shields for eye protection). For the purpose of statistical analysis, we considered each participant’s experience with a PPE item unique and independent (i.e. we did not account for the fact that the experience came from one and the same health worker). We analysed closed-ended questions with STATA 10 (StataCorp. 2007. College Station, TX) using counts, proportions, and the Chi-square test when comparisons were appropriate.

Two independent researchers analysed the answers to the open-ended questions using an iterative and reflexive process. This encompassed close reading and re-reading of the answers using constant comparison within and across different participants to identify key topics. The researchers then grouped the interpretations and understanding of the participants’ ideas and selected quotes to represent these findings, discussing discrepancies to achieve agreement.

Immediately after data collection with the Survey Monkey ^®^ instrument, all information was downloaded to an anonymized spreadsheet and removed from the online database. All analyses were performed on de-identified data.

### Informing rapid advice guideline recommendations

The rapid advice guideline was developed using the Grading of Recommendations Assessment, Development and Evaluation (GRADE) approach ^9, 10^. With this approach, clinical and public health recommendations are based on a systematic review and critical appraisal of the evidence on benefits and harms of an intervention, and an assessment of the balance between the two. Other considerations are also taken into account when an expert panel formulates recommendations, including feasibility, acceptability and resource implications of the intervention options, and the effects on equity across subpopulations. The relative value of the potential outcomes of the intervention options and the values and preferences of persons affected by the intervention are also important considerations. The findings of the survey were presented at the guideline development meeting and incorporated into evidence-to-decision tables ( Supplementary File 2) to inform the formulation of recommendations for PPE components in the context of an EVD outbreak. Evidence-to-decision tables followed the GRADE-DECIDE ^11^ approach and were populated by the WHO guideline development team in preparation for the expert panel meeting. These tables were key instruments used to present multiple sources of information to the guideline expert panel, helping to structure the discussion and to document the final judgements and decisions that underpin each recommendation.

## Results

We developed the study protocol, obtained WHO ethics approval, contacted the participants, delivered the survey, analysed the data, and presented the findings as part of the evidence-to-decision tables at the expert panel meeting where the recommendations were formulated in a period of 8 weeks.

We invited 192 health workers (166 from MSF and 26 from WHO) to participate in the survey and 74 (39%) responded. Respondents from MSF included 30 logisticians and water, sanitation and hygiene experts who were excluded because they were not part of the sampling frame. Thus 44 participants (33 physicians and 11 nurses) were included in the final analysis and their characteristics are described in Table 1.

**Table 1.** Characteristics of survey participants (n=44).

Characteristic		N (%)
Sex	Female	21 (48)
	Male	23 (52)
Age	18–24	1 (2)
	25–34	11 (25)
	35–44	20 (45)
	45–54	7 (16)
	>=55	5 (11)
Place of origin	European	24 (55)
	Americas	10 (23)
	African	5 (11)
	Asian	2 (5)
	Australia/New-Zealand	3 (7)
Organisation	MSF	37 (84)
	WHO	7 (16)
Deployment location	Sierra Leone	19 (43)
	Liberia	12 (27)
	Guinea	12 (27)
	Nigeria	1 (2)
Duration work period *	≤ 14 days	4 (10)
	15–30 days	19 (45)
	31–60 days	15 (36)
	≥ 61 days	4 (10)
Role	Physician	33 (75)
	Nurse	11 (25)
Tasks performed (more than one answer possible)	Physical examination	39 (89)
	Collection of blood samples	22 (50)
	Injections or intra-venous line insertion	37 (84)
	Collection of swabs	25 (57)
	Feeding or oral hydration	35 (80)
	Cleaning/disinfecting environment	23 (52)
	Burial	2 (5)
	Other **	21 (48)

* n=42 because of missing data for 2 survey participants. **Other tasks included: triage (n=3), medical rounds (2), nursing or direct patient care (3), outreach activities (7), checking or decontaminating colleagues (2), low risk activities such as teaching, training, administrative, pharmacy, informing family members (3), high risk activities such as carrying or lifting patients or corpses, disinfecting or spraying corpses, birth assistance, intra-osseous line insertion (7).

### PPE use and perceived risks and effects

For each of the different components of PPE, one item was used by the majority of survey participants ( Table 2). For example, 42 (95%) of participants had experience using goggles, while only seven (16%) had used a face shield (some participants had experience with both types of eye protection). Generally, health workers felt at low or extremely low risk regardless of the type of PPE used. PPE, particularly goggles, particulate respirators, and medical masks or hoods, impaired communication ( Table 2). A reduction in the ability to provide care was predominantly related to eye protection equipment - both face shields and goggles. Heat and dehydration were a significant or major issue for 31 participants using goggles (76%) compared to two (29%) using a face shield (p=0.02), and for 27 (64%) using a hood compared to none using a hair cover (p=0.02). Heat and dehydration also were a significant or major issue for the majority of individuals using a gown (n=11, 73%) or coverall (n=26, 87%); however, there was no significant difference between the two groups (p=0.41). Goggles were considered more uncomfortable (n=29, 71%) than face shields (n=2, 29%, p=0.08) ( Table 2).

**Table 2.** Health worker preferences.

	Safety		Communication		Ability to provide patient care		Personal wellbeing: heat and dehydration		Comfort	
	Extremely low risk/low risk	High risk/ extremely high risk	No or minor impairment	Impairment/ major impairment/ unable to communicate	No reduction/ minor reduction	Reduction/ major reduction/ unable to provide care	No issue/ minor issue	Significant issue/major issue/ unbearable	Comfortable/ fairly comfortable	Fairly uncomfortable/ very uncomfortable
Eye protection										
Face shield (n=7) ^†^	7 (100%)	0 (0%)	4 (57%)	3 (43%)	3 (43%)	4 (57%)	5 (71%)	2 (29%)	5 (71%)	2 (29%)
Goggles (n=42)	31/40 ^††^ (78%)	9/40 (22%)	12/41 (29%)	29/41 (71%)	8/41 (20%)	33/41 (80%)	10/41 (24%)	31/41 (76%) *	12/41 (29%)	29/41 (71%)
Nose and mouth protection										
Medical mask (n=14)	11/13 (85%)	2/12 (15%)	4/13 (31%)	9/13 (69%)	12/13 (92%)	1/13 (8%)	8/13 (62%)	5/13 (38%)	8/13 (62%)	5/13 (38%)
Respirator (n=33)	29/31 (94%)	2/31 (6%)	3/28 (11%)	25/28 (89%)	20/28 (71%)	8/28 (29%)	12/27 (44%)	15/27 (56%)	11/30 (37%)	19/30 (63%)
Gloves										
Single gloves (n=2)	0/1 (0%)	1/1 (100%)	n/a	n/a	1/1 (100%)	0/1 (0%)	1/1 (100%)	0/1 (0%)	1/1 (100%)	0/1 (0%)
Double gloves (n=44)	41/43 (95%)	2/43 (5%)	n/a	n/a	27/43 (63%)	16/43 (37%)	37/43 (86%)	6/43 (14%)	39/41 (95%)	2/41 (5%)
Rubber gloves (n=8)	6/6 (100%)	0/6 (0%)	n/a	n/a	0/3 (0%)	3/3 (100%)	2/2 (100%)	0/2 (0%)	2/3 (67%)	1/3 (33%)
Body covering										
Gown (n=16)	14/14 (100%)	0/14 (0%)	10/14 (71%)	4/14 (29%)	10/14 (71%)	4/14 (29%)	4/15 (27%)	11/15 (73%)	7/14 (50%)	7/14 (50%)
Coverall (n=31)	27/30 (90%)	3/30 (10%)	18 (58%)	13/31 (42%)	18/30 (60%)	12/30 (40%)	4/30 (13%)	26/30 (87%)	16/30 (53%)	14/30 (47%)
Head cover										
Hair cover (n=4)	4 (100%)	0 (0%)	3/3 (100%)	0/3 (0%)	3/4 (75%)	1/4 (25%)	4/4 (199%)	0/4 (0%)	4/4 (100%)	0/4 (0%)
Hood (n=42)	38/41 (93%)	3/41 (7%)	20 (48%)	22 (52%)	31/42 (74%)	11/42 (26%)	15/42 (36%)	27/42 (64%) *	27/42 (64%)	15/42 (36%)
Foot wear										
Closed shoes (n=1)	-	-	n/a	n/a	-	-	-	-		-
Rubber boots (n=44)	44 (100%)	0 (0%)	n/a	n/a	42/44 (95%)	2/44 (5%)	38/43 (78%)	5/43 (12%)	37/42 (88%)	5/42 (12%)

^†^ (n= 7) in the first column reflects the number of participants having experience with that particular item of PPE, e.g. 7 participants had used the face shield, and 42 had used goggles ^††^ The denominator in columns 2–11 reflects the number of health workers having used the item of PPE minus missing values for that specific question, e.g. only 40 out of 42 health workers who had used goggles, answered the question on safety. *Indicates p-value < 0.05. n/a, not applicable

### Experiences with PPE and suggestions for improvement

Participants indicated that fogging of goggles or face shields was a major issue, affecting visibility and potentially creating a hazard for health workers as well as patients. There was some indication that fogging was a bigger issue with goggles and a few participants indicated that they would have preferred a face shield. Two participants indicated that the goggles caused pain after using them for extended periods. A number of participants noted that goggles did not cover sufficient skin of the face and there were requests for larger goggles, which would have the added advantage of greater visibility. Other issues were the poor quality of face shield and goggles, poor fit of goggles, and the logistical challenges of waiting to clean and dry re-usable goggles. One respondent summarized it as follows: *“The goggles (are) not so comfortable and (they) felt like the "unsafe" part of the PPE. They move easily, hurt on the head, and affect vision in a negative way due to sweat, etc.”.*

Medical mask and the particulate respirator were reported to cause difficulty breathing when wet (due to sweat or condensation). One participant doubted the mask’s effectiveness when wet. Two participants were of the opinion that respirators were excessive since EVD is not airborne.

The main problem regarding gloves was the risk of having them slip down, allowing fluids to contact the skin as illustrated by the following respondent: “ *Some people found using tape over gloves (the second pair) useful as sometimes they did roll down during arduous patient care activity and in the end I also did thi*s”. Other participants also attempted to solve this problem by taping gloves to the coverall, however this occasionally resulted in the tearing of gloves or the coverall. It was also mentioned that gloves were not long enough and that they tore easily.

Many participants indicated the need for lighter suits with better ventilation. As one respondent commented: *“During the dry season and if it was a sunny day it became quickly unbearable to stay too long (in the ETU). Ebola patients need lots of care and support, full PPE hinders this process. We need lighter and cooler PPE to be able to provide better care and stay longer inside (the ETU). Full PPE causes heat exhaustion and dehydration”.* Difficulties included finding the right size coverall – in several instances the available coveralls were too small, leaving the health worker to opt for a coverall of lesser quality or have difficulties removing the coverall. A number of health workers indicated that they had difficulty taking off the coverall. Specific issues included having to remove the face shield first, leaving the eyes and face unprotected while undressing from the coverall, and problems taking off the coverall over large rubber boots. One respondent mentioned that coveralls with attached shoe covers could increase the risk of tripping. One respondent commented that boots were too big causing difficulty walking on irregular ground. As for reusable items (goggles and boots), it was mentioned that the time required to fully decontaminate and dry them sometimes brought challenges and put pressure on the team.

### Training on PPE use

A third of survey participants had received formal training over 2 to 3 days (n=15, 34%) and four (9%) reported training duration of more than 3 days. On the other hand, 20% (n=9) had received no formal or on-the-job training and another 20% (n=9) reported training for 2 hours or less. The remaining 15% of study participants (n=7) had training of one day or less. A number of participants commented that they would have liked to have had training, more formal training, or longer training. Others indicated that they would have liked to receive training before their departure, or before arriving at the treatment centre. The training topics that the survey participants would have liked included were the removal of PPE, and, how to manage eye glasses. One health worker recommended weekly refresher training, especially in the light of frequent equipment changes, which may impact the order items are put on and taken off. Another health worker commented: *“I believe that only experienced people can teach about Ebola. Teaching on the use of PPE is not about dressing and undressing. It is about using a set of behaviours with it and the understanding* of **all the underlying water and sanitation principles and applying them”.** Regarding hand hygiene, alcohol-based hand-rub was not always available and there was conflicting information in different settings about which product to use.

### Confidence using PPE and preferences

The majority of participants (n=26, 59%) were very confident that they were using PPE correctly, 17 (39%) were reasonably confident and 1 (2%) was not very confident. Generally, participants were least confident about goggles (fogging, moving/displacing), medical masks and particulate respirators (difficulty breathing, becoming uncomfortable), and gloves (rolling down, tearing). Removing PPE was also an area that people felt less confident about (e.g., taking arms and feet out of a coverall, lack of face protection during undressing if the face shield was worn outside the hood). As one health worker illustrated: *“Taking off the (Tyvek suit) coverall was difficult due to my height; it required me to wiggle out of it more than the average person”*. ** A respondent also mentioned feeling less confident working in the screening area where much lighter PPE was worn, while possibly also being exposed to infectious patients.

When asked to indicate their preference regarding two sets of PPE depicted in a picture, 8 (18%) participants preferred the PPE that was composed of lighter items, 33 (77%) participants preferred the more robust components, 2 (5%) did not have a preference and one participant did not respond to the question.

## Discussion

The 2014–2016 EVD outbreak in West Africa required extensive local and international response and for the first time since EVD was described in 1976, a large number of organizations were directly involved in clinical and laboratory activities in the field. These interactions highlighted differences in the selection and use of PPE across the organizations. Early on in the outbreak, when the cases of health worker transmission were numerous and confusion about the best available equipment was wide-spread, WHO was asked to provide technical guidance in a short period of time. When a public health emergency involves a new disease, or a known disease with a different presentation, there may be scarce or no evidence on the benefits and harms of potential interventions. Indirect evidence (e.g., from related diseases such as other blood-borne pathogens and simulation), expert opinion, and data acquired and analysed in real-time may become the best available evidence for the guideline panel. In addition, factors other than the effectiveness of interventions may have a significant influence on the direction and strength of the recommendations. Such was the situation in 2014 during the height of the EVD outbreak in West Africa; a rapid review of the effectiveness of different types of PPE for protecting health workers revealed insufficient evidence upon which to draw conclusions about optimal PPE ^8^.

In this context and within a period of 8 weeks, we developed and executed a survey, the results of which formed a critical part of the evidence upon which the recommendations developed by the expert panel were based ^12^. To the best of our knowledge, this approach of collecting primary data regarding the values and preferences of persons affected by clinical or public health recommendations in a guideline is novel in the extremely challenging setting of a public health emergency.

### Implications of the survey findings

Overall, our findings showed that health workers perceive a balance between transmission protection and the ability to effectively care for complex patients while using PPE. Health workers accept a certain degree of discomfort in return for the protection provided by PPE. The survey highlighted a slight preference of health workers for face shields compared to goggles because of less fogging, easier communication and better fit. There was no strong preference for one item of PPE over the other for all other PPE components. Given the variation in preferences for different components of PPE and the absence of data on comparative effectiveness, it may be important to provide a choice for health workers. This was, in fact, a guiding principle during the development of the PPE guidelines. Several issues raised by survey participants should be relatively straightforward to address, making a major contribution to health worker safety and comfort, such as providing a sufficient range of sizes, choice of equipment, and adequate training on how to put on and take off PPE in the conditions that will be faced in the field. Active training, in which health workers receive face-to-face training has been shown to improve doffing procedures ^13^.

### Challenges

We experienced a number of challenges planning and executing this study. We had to develop a survey questionnaire *de novo* with limited time for field testing. Although this likely had a minimal impact on the results, we noted two questions that participants appeared to have difficulty comprehending (questions 11 and 23; see Supplementary File 1); if we had had more time for field testing we could have revised the questionnaire before formal data collection began. While our aim was to include only health workers who had provided direct patient care, such as nurses and physicians, given a communication error early in the study, we invited to participate and consequently received responses from workers without direct clinical experience who had been deployed to the EVD outbreak. Because these workers were not part of our pre-defined sampling frame, we excluded their responses from the analysis. Similarly, our survey failed to take into account the fact that PPE consists of different components such as eye protection, nose and mouth protection, gloves and body coverings that work together to protect the health worker from the risk of infection. In the first part of our questionnaire we asked how the survey participant experienced individual components of PPE (e.g., goggles or face mask). However, it is difficult to review these components as isolated items, separate from the rest of the PPE. As one survey participant noted: “ *It is the combination of the respirator and the face shield which is difficult. One or the other would be manageable but both together meant major impairment*”. Another survey participant commented: “ *The coverall would probably be better tolerated if we could breathe easier and see without problems*”. In addition, although we compared gowns and coveralls, we did not specify or ask about the materials the body coverings were made of, its level of fluid resistance, or whether the head cover was attached or not. Such issues can have a significant impact on health workers’ experiences. For example, a simulation study carried out in Hong Kong in response to the outbreak of severe acute respiratory syndrome (SARS) found that PPE made of more breathable material did not lead to a significant difference in contamination but did have greater user satisfaction ^13, 14^. It also became clear that solutions to an issue with one component of PPE could compromise the safety of another element of PPE. For example, participants mentioned that they would improvise and tape gloves to the coverall in order to prevent them from slipping down, but then the coverall would tear when removing the tape. Finally, the combination of different components of PPE may change the order in which PPE items are put on and taken off, thus end-users may perform donning and doffing procedures that are different than the training they received. This is particularly relevant if there are frequent changes in the availability of specific types of PPE, as was the case early in the outbreak response.

### Study limitations

Most of the limitations of this study were caused by pragmatic decisions the research team had to make in order to complete the study in the available time. This was in and of itself an invaluable learning experience for undertaking similar projects in the future. Specifically, we had to include only international health workers deployed by WHO and MSF in our study; therefore, we did not collect information on the values and preferences of local health workers and health workers deployed by other organizations. There were two important reasons as to why we selected our sampling frame. First, we carried out the survey at the height of the EVD epidemic when local doctors and nurses were fully engaged in the response efforts and we refrained from removing them from their primary work. Internationally recruited health workers on the other hand, were usually deployed for shorter periods and could thus participate when they returned home. Second, we had little time in which to execute the survey before the guideline meeting and we anticipated that it would be a lengthier and more complex process to identify and recruit local health workers. Thus, the findings of this survey may not be applicable to local health workers. In addition, generalizability of our findings to other international health workers involved in the Ebola response may be limited due to the small size of our purposive sample.

### Study strengths

In the context of the most challenging of research settings, our study proceeded very efficiently and effectively in several regards. Peer reviewers for both the study protocol and draft survey made very helpful comments within 1 to 2 days. The WHO Ethics Review Committee approved the survey in less than two weeks. By reaching out to several key managers and opinion leaders from the two organizations, we were quickly able to identify frontline clinicians that were part of the sampling frame. The online format of the survey allowed us to quickly reach a larger number of health workers in different countries who had recent personal experience with different types of PPE in the EVD outbreak. The combination of different types of questions in our survey also worked well. Closed and Likert-scale questions made analysis of trade-offs and comparisons of health workers’ preferences possible while open-ended questions allowed the survey participants to share additional thoughts and perspectives in more depth.

## Conclusion

Our study highlights some of the challenges and potential limitations and demonstrates the feasibility of generating and incorporating primary data on end-users’ values and preferences into a rapid advice guideline developed during the height of a public health emergency with extreme field conditions. Our survey showed that health workers perceive a balance between transmission protection and their ability to effectively care for patients while wearing PPE. These findings were a critical part of the information used by the guideline development expert panel when formulating recommendations on PPE for frontline health workers caring for EVD patients in outbreak conditions.

## Ethical statement

We obtained expedited approval of the study protocol and survey from the World Health Organization Ethics Review Committee (RPC690). As approved by the ethics committee, we provided a link to the informed consent form with the survey. Participation was voluntary and implied informed consent. Participants could withdraw from the study at any time without providing any justification.

## Data availability

The data referenced by this article are under copyright with the following copyright statement: Copyright: © 2018 Den Boon S et al.

Data associated with the article are available under the terms of the Creative Commons Zero "No rights reserved" data waiver (CC0 1.0 Public domain dedication). 

Due to the small number of survey participants, the detailed information collected, and the terms in the consent form approved by the WHO Ethics Review Committee, which guaranteed participant anonymity, the individual-level data cannot be made available. Requests for raw data can be dealt with on a case-by-case basis by contacting the corresponding author Dr den Boon, who will facilitate enquiries to the WHO Ethics Review Committee.

## Supplementary material

Supplementary File 1: Study questionnaire.

Supplementary File 2: Evidence-to-decision tables used in the formulation of recommendations for the WHO Rapid Advice Guideline: Personal Protective Equipment in the context of filovirus disease outbreak response.
