# Supplementary material for: Incorporating health workers’ perspectives into a WHO guideline on personal protective equipment developed during an Ebola virus disease outbreak
Source: F1000Res. 2018 Mar 9;7:45. Originally published 2018 Jan 11. [Version 2] doi: 10.12688/f1000research.12922.2 (PMC5820616; doi:10.12688/f1000research.12922.2)
Supplement: Supplementary file 1 [file f1000research-7-15479-s0000.tgz › d1e60dc7-ba37-490b-ba3b-6a54fcc5c4ae.pdf]

Values and Preferences regarding Personal Protective Equipment in the

CONSENT FORM

You are being invited to participate in this survey on values and preferences of health care providers related to personal protective equipment (PPE) in the context of Ebola viral disease (EVD) in Africa. You are asked given your experience caring for Ebola patients in the current or previous outbreaks of the disease. This results of this survey will be used to formulate recommendations regarding the effective specifications and utilization practices for individual components or bundles of PPE, to improve safety and comfort of workers and patients in filovirus disease treatment centres.

This survey is carried out in addition to a systematic review that seeks to determine the evidence of effectiveness (benefits and harms) of double gloves, full face protection, head cover, gowns with high impermeability rating, particulate respirators, and rubber boots as PPE, when compared to alternative less robust PPE, for workers in healthcare facilities caring for patients with filovirus disease.

We are asking your consideration to participate in this survey. If you agree to participate, please continue, and fill in and submit the questionnaire. If you have any questions regarding the survey or the questionnaire, please feel free to contact the lead researcher at any time (saskiadenboon@hotmail.com). Should you not wish to continue participation after you have consented, you are free to withdraw at any time and without prejudice to you. Your participation in the survey is anonymous: your name will not appear on the questionnaire nor will it be recorded anywhere else. The database containing the questionnaire data will only be accessible by the research team. All efforts to maintain confidentiality and anonymity will be undertaken, during and following the study.

Consent for Participation
I volunteer to participate in the WHO survey on values and preferences related to PPE in the context of Ebola viral disease in Africa, conducted by the WHO department of Pandemic and Endemic Diseases. I understand that the survey is designed to gather information about experiences, and values and preferences related to different types of PPE worn by health care providers caring for patients with Ebola virus disease in Africa.

1. I will be one of approximately 20-25 people filling in the survey questionnaire. My participation in this project is voluntary.
2. I understand that I will not be paid for my participation. I may withdraw and discontinue participation at any time without penalty.
3. I understand that participation involves filling in an online questionnaire.
4. I understand that I if I fill in and submit the questionnaire, my consent is considered given.
5. I understand that the lead researcher or any researcher as part of this study, will not identify me by name in any manner, and in any reports using information obtained from this interview, and that my confidentiality as a participant in this study will remain secure.
6. I understand that this research study has been reviewed and approved by the WHO Ethics Review Board (ERC).
7. I understand that this study may be submitted for publication in the peer-reviewed literature.
8. I have read and understand the explanations/information provided to me regarding this project. I have had all my questions answered to my satisfaction, and I voluntarily agree to participate in this study. If I have any additional questions, I will communicate them to the lead researcher via e-mail.

Demographics

***1. What is your gender?**

- ◯ Female
- ◯ Male

***2. What is your age?**

- ◯ 18 to 24
- ◯ 25 to 34
- ◯ 35 to 44
- ◯ 45 to 54
- ◯ 55 to 64
- ◯ 65 to 74
- ◯ 75 or older

***3. What is your nationality?**

Your experience

***4. Where did you most recently work as a Health Care Provider with patients with Ebola virus disease?**

(province and country)

***5. When did you start work as a Health Care Provider with patients with Ebola virus disease?**

When uncertain about the exact start day, fill 01 for the day.

If you have done this work more than once, please indicate your most recent period of work.

Date / Time DD / MM / YYYY

***6. When did you stop work as a Health Care Provider with patients with Ebola virus disease?**

When uncertain about the exact stop day, fill 01 for the day.

If you have done this work more than once, please indicate your most recent period of work.

Date / Time DD / MM / YYYY

***7. What was your role /occupation?**

- physician
- nurse
- Other (please specify)

***8. Which was the organisation you worked for?**

- WHO
- MSF
- Other (please specify)

***9. Which tasks did you perform?**

(Check all that apply)

- [ ] Performing a physical examination
- [ ] Collecting blood samples
- [ ] Giving injections / inserting intra-venous line
- [ ] Taking swabs
- [ ] Feeding / orally hydrating patients
- [ ] Cleaning / disinfecting environment
- [ ] Burying dead bodies
- [ ] Other (please specify)

Your experience with Personal Protective Equipment

10. What Personal Protective Equipment were you provided with?
(If you were provided with or used different Personal Protective Equipment on different occasions, please check all that apply)

*10a. Gloves

- [ ] none
- [ ] single gloves
- [ ] double gloves
- [ ] heavy duty (rubber) gloves
- [ ] Other (please specify)

*10b. Shoes / boots

- [ ] none
- [ ] closed shoes with shoe cover
- [ ] rubber boots
- [ ] Other (please specify)

*10c. Gown

- [ ] none
- [ ] light surgical gown with impermeable apron
- [ ] impermeable gown with apron
- [ ] thick coverall (hazmat (Tyvek) suit)
- [ ] Other (please specify)

*10d. Eye protection

- [ ] none
- [ ] face shield
- [ ] goggles
- [ ] Other (please specify)

* 10e. Head cover

- [ ] none
- [ ] hair cover (cap)
- [ ] hood (full head and neck cover)
- [ ] Other (please specify)

* 10f. Respiratory protection

- [ ] none
- [ ] medical mask
- [ ] N95 respirator
- [ ] Other respirator
- [ ] Other (please specify)

Safety

Question 11 - 15 will ask about your opinion regarding safety, the ability to communicate, the ability to provide patient care, heat and dehydration, and comfort of the different aspects and types of Personal Protective Equipment. Please only provide an answer for the type of Personal Protective Equipment that you used (e.g. double gloves) - you can answer by ticking the appropriate answer box on the row "double gloves". If you did not use a certain type of PPE (e.g. not without gloves, no single gloves, no heavy duty gloves and no other type of gloves) than you can leave the row empty. If you indicated in question 10 above that you used "other" type of protection, please use the "other" option in question 11-15 to give your opinion on this item of personal protective equipment. If you used more than one type of equipment (e.g. both single and double gloves) than please provide an answer for both.

*11. Please indicate how safe you felt by ticking a box for each aspect of Personal Protective Equipment

11a. Gloves

	Extremely low risk, I felt very comfortable	Low risk, I felt comfortable	High risk, I felt uncomfortable	Extremely high risk, I felt very uncomfortable
no gloves	◯	◯	◯	◯
single gloves	◯	◯	◯	◯
double gloves	◯	◯	◯	◯
heavy duty (rubber) gloves	◯	◯	◯	◯
other	◯	◯	◯	◯

*11b. Boots

	Extremely low risk, I felt very comfortable	Low risk, I felt comfortable	High risk, I felt uncomfortable	Extremely high risk, I felt very uncomfortable
none	◯	◯	◯	◯
closed shoes with shoe covers	◯	◯	◯	◯
rubber boots	◯	◯	◯	◯
other	◯	◯	◯	◯

*11c. Gown

	Extremely low risk, I felt very comfortable	Low risk, I felt comfortable	High risk, I felt uncomfortable	Extremely high risk, I felt very uncomfortable
none	◯	◯	◯	◯
light surgical gown with impermeable apron	◯	◯	◯	◯
impermeable gown with apron	◯	◯	◯	◯
thick coverall (hazmat (Tyvek) suit)	◯	◯	◯	◯
other	◯	◯	◯	◯

*11d. Eye protection

	Extremely low risk, I felt very comfortable	Low risk, I felt comfortable	High risk, I felt uncomfortable	Very high risk, I felt very uncomfortable
none	◯	◯	◯	◯
face shield	◯	◯	◯	◯
goggles	◯	◯	◯	◯
other	◯	◯	◯	◯

*11e. Head cover

	Extremely low risk, I felt very comfortable	Low risk, I felt comfortable	High risk, I felt uncomfortable	Very high risk, I felt very uncomfortable
none	◯	◯	◯	◯
hair cover	◯	◯	◯	◯
hood	◯	◯	◯	◯
other	◯	◯	◯	◯

*11f. Respiratory protection

	Extremely low risk, I felt very comfortable	Low risk, I felt comfortable	High risk, I felt uncomfortable	Extremely high risk, I felt very uncomfortable
none	◯	◯	◯	◯
medical mask	◯	◯	◯	◯
N95 respirator.	◯	◯	◯	◯
other respirator	◯	◯	◯	◯
other	◯	◯	◯	◯

11g. Comments

Communication

12. To what extent did each of the items affect your ability to communicate with patients?

12a. Gown

	No impairment of communication	Minor impairment of communication	Impairment of communication but manageable	Major impairment of communication	Unable to communicate
none	○	○	○	○	○
light surgical gown with impermeable apron	○	○	○	○	○
impermeable gown with apron	○	○	○	○	○
thick coverall (hazmat (Tyvek) suit)	○	○	○	○	○
other	○	○	○	○	○

* 12b. Eye protection

	No impairment of communication	Minor impairment of communication	Impairment of communication but manageable	Major impairment of communication	Unable to communicate
none	○	○	○	○	○
face shield	○	○	○	○	○
goggles	○	○	○	○	○
other	○	○	○	○	○

* 12c. Head cover

	No impairment of communication	Minor impairment of communication	Impairment of communication but manageable	Major impairment of communication	Unable to communicate
none	○	○	○	○	○
hair cover	○	○	○	○	○
hood	○	○	○	○	○
other	○	○	○	○	○

***12d. Respiratory protection**

	No impairment of communication	Minor impairment of communication	Impairment of communication but manageable	Major impairment of communication	Unable to communicate
none	◯	◯	◯	◯	◯
medical mask	◯	◯	◯	◯	◯
N95 respirator.	◯	◯	◯	◯	◯
other respirator	◯	◯	◯	◯	◯
other	◯	◯	◯	◯	◯

12e. Comments on Personal Protective Equipment hindering or facilitating communication:

Ability to provide patient care

*13. To what extent did each protective item impair your ability to provide effective patient care, including your ability to perform tasks requiring manual dexterity, e.g. giving an injection, finding a vein??

13a. Gloves

	No reduction in ability to provide care	Minor reduction in ability to provide care	Important reduction in ability to provide care, but manageable	Major reduction in ability to provide care	Unable to provide care
no gloves	◯	◯	◯	◯	◯
single gloves	◯	◯	◯	◯	◯
double gloves	◯	◯	◯	◯	◯
heavy duty (rubber) gloves	◯	◯	◯	◯	◯
other	◯	◯	◯	◯	◯

*13b. Boots

	No reduction in ability to provide care	Minor reduction in ability to provide care	Important reduction in ability to provide care, but manageable	Major reduction in ability to provide care	Unable to provide care
none	◯	◯	◯	◯	◯
closed shoes with shoe covers	◯	◯	◯	◯	◯
rubber boots	◯	◯	◯	◯	◯
other	◯	◯	◯	◯	◯

*13c. Gown

	No reduction in ability to provide care	Minor reduction in ability to provide care	Important reduction in ability to provide care, but manageable	Major reduction in ability to provide care	Unable to provide care
none	◯	◯	◯	◯	◯
light surgical gown with impermeable apron	◯	◯	◯	◯	◯
impermeable gown with apron	◯	◯	◯	◯	◯
thick coverall (hazmat (Tyvek) suit)	◯	◯	◯	◯	◯
other	◯	◯	◯	◯	◯

*13d. Eye protection

	No reduction in ability to provide care	Minor reduction in ability to provide care	Important reduction in ability to provide care, but manageable	Major reduction in ability to provide care	Unable to provide care
none	○	○	○	○	○
face shield	○	○	○	○	○
goggles	○	○	○	○	○
other	○	○	○	○	○

*13e. Head cover

	No reduction in ability to provide care	Minor reduction in ability to provide care	Important reduction in ability to provide care, but manageable	Major reduction in ability to provide care	Unable to provide care
none	○	○	○	○	○
hair cover	○	○	○	○	○
hood	○	○	○	○	○
other	○	○	○	○	○

*13f. Respiratory protection

	No reduction in ability to provide care	Minor reduction in ability to provide care	Important reduction in ability to provide care, but manageable	Major reduction in ability to provide care	Unable to provide care
none	○	○	○	○	○
medical mask	○	○	○	○	○
N95 respirator.	○	○	○	○	○
other respirator	○	○	○	○	○
other	○	○	○	○	○

13g. Comments

Personal wellbeing

***14. To what extent did each protective item affect your personal wellbeing with regards to heat and dehydration?**

14a. Gloves

	Heat and dehydration was not an issue	Heat and dehydration was a minor issue	Heat and dehydration was a significant issue	Heat and dehydration were a major issue	Heat and dehydration became unbearable very quickly
no gloves	○	○	○	○	○
single gloves	○	○	○	○	○
double gloves	○	○	○	○	○
heavy duty (rubber) gloves	○	○	○	○	○
other	○	○	○	○	○

*14b. Boots

	Heat and dehydration was not an issue	Heat and dehydration was a minor issue	Heat and dehydration was a significant issue	Heat and dehydration were a major issue	Heat and dehydration became unbearable very quickly
no particular shoe provided	○	○	○	○	○
closed shoes with shoe cover	○	○	○	○	○
rubber boots	○	○	○	○	○
other	○	○	○	○	○

*14c. Gown

	Heat and dehydration was not an issue	Heat and dehydration was a minor issue	Heat and dehydration was a significant issue	Heat and dehydration were a major issue	Heat and dehydration became unbearable very quickly
none	○	○	○	○	○
light surgical gown with impermeable apron	○	○	○	○	○
impermeable gown with apron	○	○	○	○	○
thick coverall (hazmat (Tyvek) suit)	○	○	○	○	○
other	○	○	○	○	○

* 14d. Eye protection

	Heat and dehydration was not an issue	Heat and dehydration was a minor issue	Heat and dehydration was a significant issue	Heat and dehydration were a major issue	Heat and dehydration became unbearable very quickly
none	◯	◯	◯	◯	◯
face shield	◯	◯	◯	◯	◯
goggles	◯	◯	◯	◯	◯
other	◯	◯	◯	◯	◯

* 14e. Head cover

	Heat and dehydration was not an issue	Heat and dehydration was a minor issue	Heat and dehydration was a significant issue	Heat and dehydration were a major issue	Heat and dehydration became unbearable very quickly
none	◯	◯	◯	◯	◯
hair cover	◯	◯	◯	◯	◯
hood	◯	◯	◯	◯	◯
other	◯	◯	◯	◯	◯

* 14f. Respiratory protection

	Heat and dehydration was not an issue	Heat and dehydration was a minor issue	Heat and dehydration was a significant issue	Heat and dehydration were a major issue	Heat and dehydration became unbearable very quickly
none	◯	◯	◯	◯	◯
medical mask	◯	◯	◯	◯	◯
N95 respirator	◯	◯	◯	◯	◯
other respirator	◯	◯	◯	◯	◯
other	◯	◯	◯	◯	◯

* 14g. How long, on average, could you wear the PPE?

◯ 30 minutes or less

◯ between 30 minutes and 1 hour

◯ between 1 and 2 hours

◯ more than 2 hours

14h. Comments on heat, dehydration and how it impacted the duration you could wear the PPE.

Comfort

*15. How comfortable did you find each of the following protective items?

15a. Gloves

	Comfortable to wear	Fairly comfortable to wear	Fairly uncomfortable to wear	Very uncomfortable to wear
no gloves	○	○	○	○
single gloves	○	○	○	○
double gloves	○	○	○	○
heavy duty (rubber) gloves	○	○	○	○
other	○	○	○	○

*15b. Boots

	Comfortable to wear	Fairly comfortable to wear	Fairly uncomfortable to wear	Very uncomfortable to wear
none	○	○	○	○
closed shoes with shoe cover	○	○	○	○
rubber boots	○	○	○	○
other	○	○	○	○

*15c. Gown

	Comfortable to wear	Fairly comfortable to wear	Fairly uncomfortable to wear	Very uncomfortable to wear
none	○	○	○	○
light surgical gown with impermeable apron	○	○	○	○
impermeable gown with apron	○	○	○	○
thick coverall (hazmat (Tyvek) suit)	○	○	○	○
other	○	○	○	○

*15d. Eye protection

	Comfortable to wear	Fairly comfortable to wear	Fairly uncomfortable to wear	Very uncomfortable to wear
none	○	○	○	○
face shield	○	○	○	○
goggles	○	○	○	○
other	○	○	○	○

*15e. Head cover

	Comfortable to wear	Fairly comfortable to wear	Fairly uncomfortable to wear	Very uncomfortable to wear
none	◯	◯	◯	◯
hair cover	◯	◯	◯	◯
hood	◯	◯	◯	◯
other	◯	◯	◯	◯

*15f. Respiratory protection

	Comfortable to wear	Fairly comfortable to wear	Fairly uncomfortable to wear	Very uncomfortable to wear
none	◯	◯	◯	◯
medical mask	◯	◯	◯	◯
N95 respirator	◯	◯	◯	◯
other respirator	◯	◯	◯	◯
other	◯	◯	◯	◯

Appearance, improvements, training, hand hygiene, and difficulties

16. Do you have additional considerations or comments related to a specific item of Personal Protective Equipment you were wearing that were not asked above? *(please specify the Personal Protective Equipment you are commenting on)*

***17. The effect of my appearance on my relations with patients, family members and the community was:**

- Null
- Minor
- Moderate
- Substantial
- Severe

Comments

18. In your opinion, what improvements could be made regarding personal protective equipment?

***19. Can you describe the training you received on Personal Protective Equipment?**

19a. Who provided the training?

Provide here the job title of the person providing the training, and/or the organisation he/she was from - do not give a name

***19b. How long lasted the training?**

***19c. Did the training involve the following?**

(tick all that apply)

- [ ] general Infection Prevention and Control measures
- [ ] mode of transmission of Ebola
- [ ] where and when to wear PPE
- [ ] putting on PPE (theory)
- [ ] putting on PPE (practical exercise)
- [ ] taking off PPE (theory)
- [ ] taking off PPE (practical exercise)
- [ ] working in pairs
- [ ] changing gloves between patients
- [ ] avoiding touching mouth and eyes with hands
- [ ] disinfection and cleaning of Personal Protective Equipment
- [ ] hand hygiene
- [ ] hazard awareness
- [ ] Other (please specify)

19d. If you could change one aspect of the training you had, what would it be?

*** 20a.What type of hand hygiene was recommended?**

- ◯ no particular recommendation
- ◯ hand washing with water and soap, before and/or after wearing gloves
- ◯ hand washing with diluted chlorine
- ◯ disinfection with an alcohol based solution
- ◯ Other (please specify)

*** 20b. Which products were available for hand hygiene?**

tick all that apply

- ☐ none
- ☐ water and soap
- ☐ diluted chlorine solution
- ☐ alcohol based solution
- ☐ Other (please specify)

*** 20c. How often did you follow the hand hygiene protocol?**

- ◯ consistently
- ◯ usually
- ◯ sometimes
- ◯ rarely
- ◯ never

20d. Please provide any comments on how you experienced the hand hygiene protocol, e.g what you liked or did not like about it and what you would have liked to change.

***21a. How confident were you that you were using the Personal Protective Equipment correctly (including removing PPE)?**

- Very confident
- Reasonably confident
- Not very confident
- Not confident at all

21b. What did you feel the least confident about (which item, or which aspects of their use)?

***22a. Did you experience any difficulties or accidents while removing your Personal Protective Equipment?**

- No
- Yes

22b. If your answer was "yes", can you describe those difficulties or accidents?

Values and Preferences regarding Personal Protective Equipment in the

Values and Preferences regarding Personal Protective Equipment in the

Here is a picture of two different types of Personal Protective Equipment currently used. The Personal Protective Equipment on the left (A) consists of single (or double) gloves, a face shield, a single mask, and a surgical impermeable gown with impermeable apron. The Personal Protective Equipment on the right (B) consists of double gloves, goggles and a mask and full sealing of the face, a double mask and a heavy impermeable gown and apron.

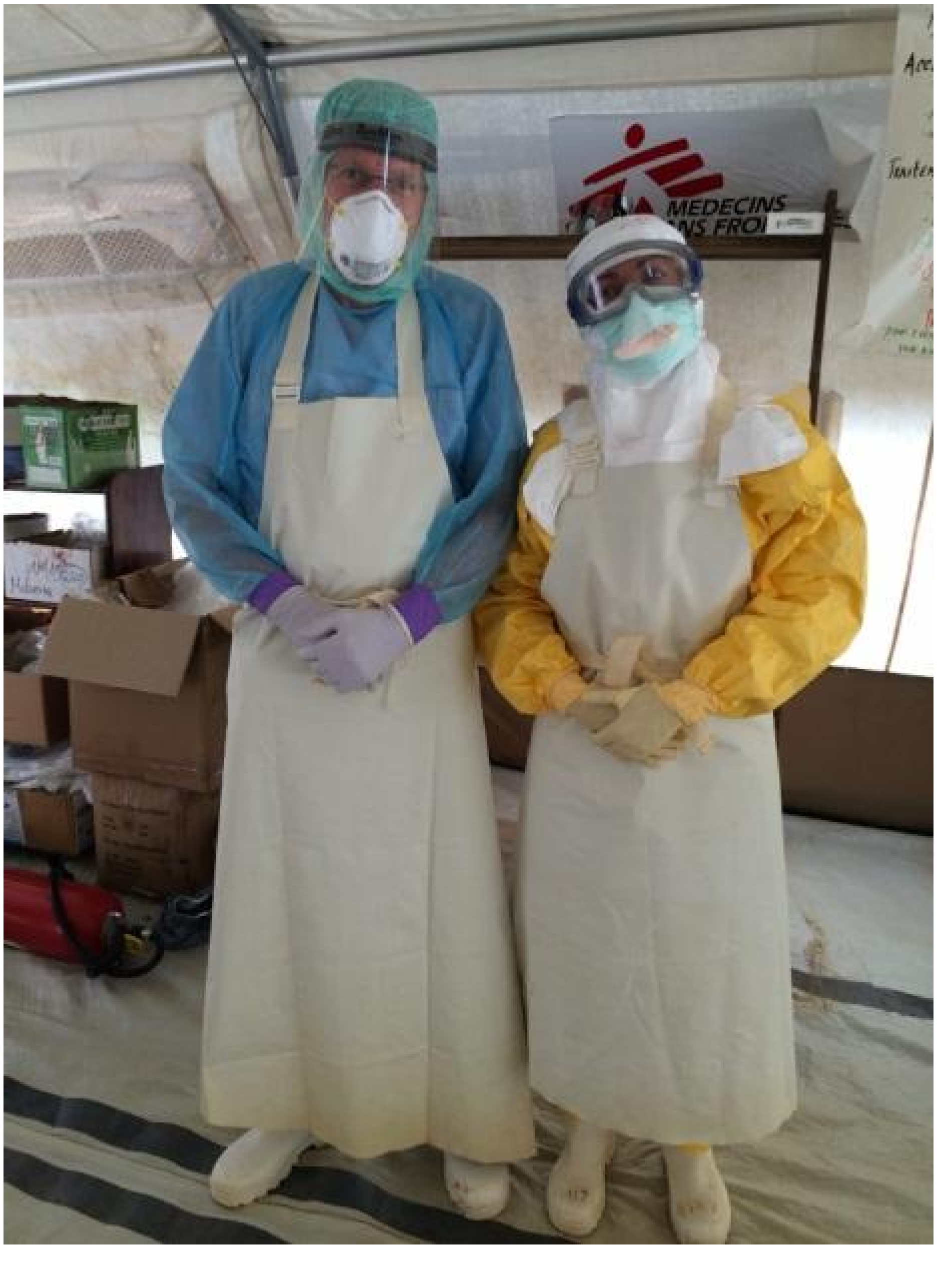

23. Based on your own experiences and risk perceptions, which type of Personal Protective Equipment would you prefer to wear when caring for patients with Ebola virus disease?

- ○ Strongly prefer A
- ○ Some preference for A
- ○ No preference between A or B
- ○ Some preference for B
- ○ Strongly prefer B

Thank you very much for taking the time to fill in this questionnaire. We will send you a summary of the survey findings as soon as they are available.

Please press the “done” button to submit your questionnaire.
