# Supplementary material for: Incorporating health workers’ perspectives into a WHO guideline on personal protective equipment developed during an Ebola virus disease outbreak
Source: F1000Res. 2018 Mar 9;7:45. Originally published 2018 Jan 11. [Version 2] doi: 10.12688/f1000research.12922.2 (PMC5820616; doi:10.12688/f1000research.12922.2)
Supplement: Supplementary file 2 [file f1000research-7-15479-s0001.tgz › 87a4a114-2c12-48e6-8d77-b6d6a6a90225.docx]

Supplementary File 2. Evidence-to-decision tables.

These tables were presented to the Guideline Development Group on 6 and 7 October 2014, at which point 38 health workers had responded to the survey on values and preferences. After the technical consultation, an additional six health workers responded, giving a total of 44 survey participants (as noted in the main text of the guideline). The six additional responses did not make a difference to the survey results.

| **Eye protection** | |
| --- | --- |
| Background | Human-to-human transmission of filovirus results from contact between broken skin or mucous membranes, such as in the mouth, nose and eyes, and virus-containing body fluids of a symptomatic infected person. It is therefore important to protect the eyes from coming into contact with such body fluids. |
| PICO | What are the benefits and harms of full-face protection compared with goggles for health workers caring for patients with filovirus disease?  **Population:** health workers in health care facilities  **Intervention:** goggles  **Comparator:** face shield  **Outcomes:** see below |
| Evidence of effectiveness: desirable effects | **Outcome 1:** prevention of virus transmission to health care provider  No comparative evidence. No estimate of effectiveness. |
| Evidence of effectiveness: undesirable effects | **Outcome 2:** fogging, reduced visibility.  No evidence found.  **Outcome 3:** inadvertent touching of face  No evidence found |
| Values and preferences | **Literature review**  One study found that barriers to use of protective eyewear, in general, included complaints of somatic effects (headaches and dizziness), interference with prescribed eyewear, and impaired vision related to fogging and scratches on the eyewear. A study reporting on an Ebola outbreak found that face shields were preferred to goggles by community members because they were considered less frightening, since they allowed the health worker to be recognized. They were also preferred by the health workers involved in an Ebola outbreak because they were thought to: (1) offer better protection, by covering the nose and mouth; (2) be more comfortable; and (3) fog less easily than goggles.  **Survey questionnaire**  Of 38 respondents, 7 had experience with face shields and 36 with goggles. Of those using goggles, 8 (22%) felt at high risk or extremely high risk compared with no one in the group using the face shield. Nine of those using goggles (25%) considered them to be a major impairment to communication compared with none in the group using face shields. In the goggles group, 75% thought there was an important or major reduction in their ability to provide patient care compared with 47% in the face shield group. Personal discomfort (heat stress and dehydration) was a major issue or unbearable for 12 (34%) of the respondents in the goggles group compared with none in the face shield group. Six people in the goggles group (17%) reported that the goggles were very uncomfortable, compared with none in the face shield group.  In summary, a greater proportion of goggle wearers than face shield wearers felt at increased risk. Goggles were also considered less comfortable, led to lower levels of well-being, and reduced ease of communication and of providing patient care.  **Issues mentioned**   - The biggest problem with the goggles was fogging. Fogging may increase the risk of accidental exposure to virus, reduce ability to provide patient care, and reduce the time that can be spent in the high-risk area. - Goggles were the wrong size or fitted badly. - Goggles were of poor quality. - Goggles moved easily or slid off. - Difficulty of wearing goggles together with glasses. - The fact that the face shield is worn outside the hood resulted in lower eye and face protection when the suit was being removed. |
| Resource use | Average cost of goggles, US$ 4.3  Average cost of disposable full-face shield, US$ 2.70 |
| Feasibility | Both options seem feasible. Consideration should be given to how face shields and goggles can be combined with other elements of PPE (e.g. do goggles prevent a good fit of the respirator, should face shields be worn over or under a head cover, etc.)  It might be difficult to combine goggles with prescription glasses. |
| Applicability | This recommendation is applicable to health workers involved in caring for patients suffering from filovirus disease. |
| Implementation considerations | - Specifications - Anti-fogging product (anti-fog spray) may be useful when using goggles - Training in use of PPE - Quality criteria - WHO to develop training materials for PPE use - WHO to develop protocols for safe disposal or decontamination prior to reuse, as appropriate |
| Research priorities | Surveys of barriers to use, effectiveness studies comparing different products. |

| **Nose and mouth protection** | |
| --- | --- |
| Background | Filovirus infection can be transmitted by infectious droplets coming into contact with the mucosa of the nose and mouth. Appropriate protection is needed for these surfaces. |
| PICO | What are the benefits and harms of particulate respirators compared with medical or surgical masks for health care workers caring for patients with filovirus disease in health care facilities?  **Population:** health workers in health care facilities  **Intervention:** particulate respirators (N95 or equivalent mask) for use by staff for whom the respirators have been fit-tested, who are medically cleared and trained.  **Comparator:** medical (surgical) mask.  **Outcomes:** see below |
| Evidence of effectiveness: desirable effects | **Outcome 1:** prevention of virus transmission to health care providers  No comparative evidence. No estimate of effectiveness  **Outcome 2:** transmission of virus to and between patients  No comparative evidence. No estimate of effectiveness. |
| Evidence of effectiveness: undesirable effects | **Outcome 3:** comfort and dexterity with use under conditions of high ambient temperature.  No comparative evidence. No estimate of effectiveness. |
| Values and preferences | **Literature review**  Factors that were reported to negatively influence acceptability of medical masks and respirators included increased fatigue, impaired critical mental ability, discomfort, anxiety of the user, and difficulty communicating. In general, surgical masks were preferred over respirators, because of their greater usability and lower associated discomfort, fatigue and odour; however, they offer limited protection during aerosol-generating procedures. Warmth and wetness around the face were often cited as a problem when using particulate respirators and can lead to increased anxiety for the user. However, there are some ways of decreasing the heat burden of protective facemasks, including the promotion of nasal breathing and the use of exhalation valves.  **Survey questionnaire**  Ten survey respondents had experience using medical masks and 30 using N95 respirators. Two participants who had used an N95 mask felt at high risk or extremely high risk; all other respondents (both medical mask and N95 respirator) felt at low or extremely low risk.  **Communication impairment:** this was considered to be more of a problem with the N95 respirator:   - no or minor impairment: medical mask, 33%; N95 respirator, 11%; - major impairment: medical mask, 11%; N95 respirator, 39%.   **Ability to provide patient care:** this was more reduced for the N95 respirator:   - no or minor reduction: medical mask, 100%; N95 respirator, 70%; - important or major reduction: medical mask, 0%; N95 respirator, 30%.   **Personal well-being (heat stress and dehydration):** perceived to be a greater issue for the N95 respirator:   - no or minor issue: medical mask, 75%; N95 respirator, 48%; - significant or major issue or unbearable: medical mask, 25%; N95 respirator, 52%.   **Comfort:** N95 mask was reported as less comfortable:   - comfortable or fairly comfortable: medical mask, 76%; N95 respirator, 39%; - uncomfortable or fairly uncomfortable: medical mask, 25%; N95 respirator, 61%.   Issues mentioned   - Respondents found it hard to breathe when the mask or respirator was wet with condensation. - Two respondents thought that the N95 was excessive for Ebola. - There was an impact on communication, both verbal and non-verbal. |
| Resource use | Respirators, half-sphere, duckbill or folded (N95/FFP2), US$ 1.53  Mask, surgical with splash resistance, flat, rectangular with folds, US$ 0.01 |
| Feasibility | Feasibility, both in terms of availability and acceptability to users, needs to be considered. |
| Applicability | This recommendation is applicable to health workers involved in caring for patients suffering from filovirus disease. |
| Implementation considerations | - Training for health workers in appropriate use - Provisions for ensuring continuous availability of items for use - Protocol for reuse of items or waste disposal, as appropriate - WHO to develop training materials on use - WHO to develop recommendations for disposal |
| Research priorities | Comparison of masks with respirators and other alternatives, cross-sectional studies in different settings to understand protective effects, compliance surveys, perceptions of barriers.  Further research into mode of transmission of Ebola: can Ebola be transmitted via airborne particles? |

| **Gloves** | |
| --- | --- |
| Background | Human-to-human transmission of filovirus results from contact between broken skin or mucous membranes, such as in the mouth, nose and eyes, and virus-containing body fluids of a symptomatic infected person. Gloves prevent the hands becoming contaminated. Proper use of gloves will help to prevent transmission via the hands to other parts of the body of the carer, to other patients and to the environment. |
| PICO | What are the benefits and harms of double gloves or heavy-duty rubber gloves compared with single gloves for health workers caring for patients with filovirus disease?  **Population:** health workers in health care facilities  **Intervention 1:** double gloves  **Intervention 2:** heavy-duty rubber gloves  **Comparator:** single gloves  **Outcomes:** see below |
| Evidence of effectiveness: desirable effects | **Outcome 1:** prevention of virus transmission to health care provider  No comparative evidence. No estimate of effectiveness.  **Outcome 2:** prevention of transmission of the virus to and between patients  No comparative evidence. No estimate of effectiveness. |
| Evidence of effectiveness: undesirable effects | **Outcome 3:** glove perforation  No comparative evidence. No estimate of effectiveness.  **Outcome 4:** manual dexterity of the user  No evidence available  **Outcome 5:** tactile sensitivity  No evidence available |
| Values and preferences | **Literature review** (not restricted to filovirus or haemorrhagic fevers)  The majority of reports relating to double-gloving focus on the use of double gloves in surgical wards. These reports have identified decreased tactile sensation, impaired dexterity, and discomfort as the main issues related to double-gloving. One study found that, in most cases, after two days, surgeons no longer had a feeling of impaired tactile sensation when using double gloves. In two studies, surgeons preferred using larger gloves on the inside and “normal” size gloves on the outside.  **Response to survey questionnaire**  All 38 respondents had experience with double-gloving, one respondent had experience with single gloves and six with rubber gloves.  The one person who had experience with both single- and double-gloving felt at high risk with the single gloves and at low risk with the double gloves, and experienced no difference in heat stress, comfort or ability to provide care.  **Perception of risk:** extremely low or low: double gloves, 97%; rubber gloves, 100%.  **Dexterity while providing care:** double gloves: no or minor reduction, 68%; important or major reduction, 32%.  **Personal discomfort due to heat and dehydration:** double gloves: no or minor issue, 84%; significant issue, 16%.  **Comfort:** double gloves: comfortable or fairly comfortable, 94%.  Issues mentioned with regard to single- and double-gloving:   - Quality of the gloves. - Gloves not strong enough, thus tearing easily. - Gloves not long enough, or tending to slide down, exposing skin. - Rubber gloves: hard to see whether there are holes. - Gloves (including rubber gloves) more friable when being removed as a result of exposure to chlorine solution. - Gloves were frequently mentioned as the item of PPE that health workers felt least confident about. |
| Resource use | Double gloves are more expensive than single gloves (but not twice as expensive, because inner glove may be changed less frequently). |
| Feasibility | The options of single and double gloves are both feasible to implement.  Rubber gloves are more likely to influence dexterity during patient care; quality is widely variable between manufacturers and difficult to control; discarding after use (waste management) will pose problems. |
| Applicability | This recommendation is applicable to health workers in direct contact with patients suffering from filovirus disease. |
| Implementation considerations | Hand hygiene should be performed according to WHO guidelines (6).  Gloves should be used in combination with other elements of PPE and in compliance with general infection prevention and control measures.  Ensure safety of injections and phlebotomy procedures and management of sharps.  Use correctly sized gloves.  All gloves should comply with quality criteria.  As recommended in the WHO guidance mentioned above, gloves should be put on when the health worker enters the patient care area. They should be changed between tasks and procedures on the same patient after contact with potentially infectious material. They should also be changed if heavily soiled with blood or any body fluids, or when they are torn or damaged. Gloves should be removed after use, before the health worker touches non-contaminated items and surfaces, and before going to another patient. Careful hand hygiene should always be performed immediately after removal.  Since 4–17% of health workers have an allergic reaction to latex, gloves made of other materials should be available.  Health workers should be trained in putting on and taking off the recommended PPE, including gloves. |
| Research priorities | Comparative studies on different glove materials, observational studies of compliance, staff surveys of perceived comfort, barriers to compliance, innovative low-cost materials for elbow-length gloves. |

| **Gown or coverall** | |
| --- | --- |
| Background | Splashing of contaminated fluids onto non-intact skin surfaces can transmit filovirus. |
| PICO | What are the benefits and harms of highly impermeable gowns compared with other items that cover exposed skin?  **Population:** health workers caring for patients with filovirus disease in health care facilities  **Intervention:** impermeable gown  **Comparator 1:** surgical gown  **Comparator 2:** coverall  **Outcomes:** see below |
| Evidence of effectiveness: desirable effects | **Outcome 1:** prevention of virus transmission to health care provider  No comparative evidence. No estimate of effectiveness. |
| Evidence of effectiveness: undesirable effects | **Outcome 2:** personal well-being (heat stress, dehydration, hyperthermia, heat stroke, pre-syncope and syncope)  No comparative evidence. No estimate of effectiveness.  **Outcome 3:** dexterity, ability to perform procedures and tasks, and ability to move  No comparative evidence. No estimate of effectiveness.  **Outcome 4:** maximum tolerated time to wear the equipment and thus be available to care for patients.  No comparative evidence. No estimate of effectiveness. |
| Values and preferences | **Literature review**  Interference with work activities and heat stress were cited as issues related to the use of impermeable gowns and impermeable suits or coveralls. The MSF guidelines report that gowns are more comfortable as long as there is limited bending and lifting, and that gowns are more acceptable in environments where it is culturally inappropriate for women to wear trousers. Coveralls allow easier movement than surgical gowns; however, both may pose a significant threat of hyperthermia. One way of combating the risk of hyperthermia is to limit the amount of time an individual wears impermeable clothing. A literature review by Health Sciences Laboratory suggested that the tolerance time for individuals wearing protective coveralls and engaging in moderate physical activity at 20 °C is approximately two hours; however, most of the studies reviewed did not take into account other factors that contribute to heat stress, such as the use of a respirator.  **Survey questionnaire**  Most survey participants (28) had experience with coveralls, followed by impermeable gowns (12) and surgical gowns (2). Five respondents mentioned the use of other types of gown, including aprons, disposable aprons, and a yellow hazardous material suit with thick apron.  **Risk of transmission:** survey participants generally felt at low or very low risk, irrespective of the gown they were wearing (coverall, 88%; impermeable gown and surgical gown, 100%).  **Communication:** this was more frequently considered to be impaired when using the coverall (42%) than the impermeable gown (18%), as was ability to provide patient care (41% vs 27 %).  **Personal well-being (heat stress and dehydration) and comfort:** there was considerable variability among health workers for both the coverall and the impermeable gown.  **Quality and requested specifications of the gown**  A number of respondents commented that the coveralls and suits were often too small, leading to potentially dangerous situations, such as exposed skin and difficulty undressing. One participant mentioned that a thumb or finger loop with elastic at the hand-opening should be used to keep the suit in place, otherwise gloves tended to slip out of the suit because of sweat. One participant mentioned that metal hooks and clip systems on the aprons should be avoided (also because of possible tearing of gloves) and that slipknots were the easiest to undo. Suits with attached foot covers were thought to be a problem, because the foot covers were much longer than the boots, causing risk of tripping. |
| Resource use | Average cost of coveralls with elastic wrists, ankles, hood, hidden zipper, disposable:  type 3, US$ 11.37  type 4, US$ 5.34  type 5 and 6, US$ 5.00  Average cost of reusable heavy duty aprons, US$ 7 |
| Feasibility | Heat and humidity severely reduce the time that health workers can wear the coveralls, especially the type 3 coverall. |
| Applicability | This recommendation is applicable to health workers involved in caring for patients suffering from filovirus disease. |
| Implementation considerations | - Specifications - Training in use - Quality criteria - Protocols for reuse or disposal - Different organizations have a preference for a different types of coverall, which may lead to confusion among users - Putting on and taking off |
| Research priorities | There is a strong need for research on PPE materials. Suits that are lighter but stronger and that allow heat exchange (moisture evaporation, ventilation system) are urgently needed. Research is also needed on how suits can provide more integrated protection, e.g. by including a hood and mouth protection. |

| **Boots** | |
| --- | --- |
| Background | Human-to-human transmission of filovirus results from contact between broken skin or mucous membranes, such as in the mouth and eyes, and virus-containing body fluids of a symptomatic infected person. People suffering from Ebola virus disease often have diarrhoea, vomiting and haemorrhage, leading to contamination of floors and other surface areas with faeces, vomit and blood. Solid footwear is therefore an important part of any PPE used by health workers in contact with Ebola patients. |
| PICO | What are the benefits and harms of rubber boots compared with closed shoes with or without shoe covers for health workers caring for patients with filovirus disease?  **Population:** health workers caring for patients with filovirus disease in health care facilities  **Intervention:** rubber boots  **Comparator:** closed shoes with or without shoe cover  **Outcomes:** see below |
| Evidence of effectiveness: desirable effects | **Outcome 1:** prevention of virus transmission to health care providers  No comparative evidence. No estimate of effectiveness.  **Outcome 2:** prevention of transmission of the virus to and between patients  No comparative evidence. No estimate of effectiveness. |
| Evidence of effectiveness: undesirable effects | **Outcome 3:** increased body temperature  No evidence available.  **Outcome 4:** difficulty in movement  No evidence available. |
| Values and preferences | **Literature review**  There is little documentation on the preference of health workers for rubber boots or shoe covers. However, one study found that laundry workers in a hospital preferred not to use boots as they were reported to be ill-fitting (too large, especially for women) and uncomfortable, and slowed the workers’ movements during work activities. One individual with experience in the Ebola outbreak reported that gumboots were preferable, as workers had to balance on one foot to remove boot covers.  **Survey questionnaire**  Only one person had experience with closed shoes; the other 38 survey participants wore boots. The person who had worn closed shoes did not comment on perceived safety, personal well-being, comfort or the impact on being able to provide patient care.  Those wearing boots provided the following answers:  **Safety:** low or very low risk, 38 (100%)  **Ability to provide patient care:** no or minor reduction, 36 (95%); important reduction, 2 (5%)  **Personal well-being (heat stress and dehydration):** no or minor issue, 33 (89%); significant issue, 4 (11%)  **Comfort:** comfortable or fairly comfortable, 32 (89%); fairly uncomfortable or uncomfortable, 4 (11%)  **Some issues mentioned**   - Boots are sometimes too big or a poor fit, and not enough sizes are available. Big boots are clumsy and increase the risk of tripping. - It is difficult to remove the coverall over the rubber boots: coveralls could get stuck on the rubber boots during removal; if the extremes of the coverall legs are too loose, they could be dragged under the boots; there is a risk of touching the boots with hands. - There is a preference for not re-using boots, or for having a personal pair. - Time is needed to decontaminate reusable items, including boots, which may not be dry when needed. - Foot covers attached to suits were much longer than the boots, and hung 5–8 cm beyond the toes, leading to a risk of tripping. |
| Resource use | Boots, rubber, pair, any colour, US$ 12  Shoe covers, no information available (may sometimes be attached to the coverall)  Closed shoes, no information available (these should be strong closed shoes, that should only be worn while caring for patients in the health centre, and should not be worn outside the health centre) |
| Feasibility | Both options seem feasible. Boots are most commonly used and well tolerated, provided that sufficient sizes are available. |
| Applicability | This recommendation is applicable to health workers involved in caring for patients suffering from filovirus disease. |
| Implementation considerations | - Specifications - Training of staff in proper use - Protocol for decontamination, storage and reuse - Making available sufficient numbers for all staff needing PPE - WHO should develop specifications and training materials on the use of PPE by health workers |
| Research priorities | Surveys of perceived barriers to compliance, cross-sectional studies on protection afforded. |

| **Head cover** | |
| --- | --- |
| Background | Human-to-human transmission of filovirus results from contact between broken skin or mucous membranes, such as in the mouth and eyes, and virus-containing body fluids of a symptomatic infected person. A head cover will help protect the scalp from exposure to such body fluids. |
| PICO | What are the benefits and harms of a hood compared with a hair cover for health workers caring for patients with filovirus disease?    **Population:** health workers in health care facilities  **Intervention:** head cover or hood  **Comparator 1**: hair cover  **Comparator 2:** no head cover.  **Outcomes:** see below |
| Evidence of effectiveness: desirable effects | **Outcome 1:** prevention of virus transmission to health care providers  No comparative evidence. No estimate of effectiveness.  **Outcome 2:** prevention of transmission of the virus to and between patients  No comparative evidence. No estimate of effectiveness. |
| Evidence of effectiveness: undesirable effects | **Outcome 3:** discomfort and heat affecting performance  No comparative evidence. No estimate of effectiveness. |
| Values and preferences | **Literature review**  This was not included in the literature review.  **Survey questionnaire**  Four participants had experience of wearing a hair cover, and 36 had experience of wearing a hood.  The four participants who had worn a hair cover felt at low or very low risk. The hair cover did not impair communication. One of the four participants thought the hair cover led to an important reduction in patient care, while the other three thought there was no reduction. The hair cover was thought to be comfortable or fairly comfortable by all and did not lead to a reduction in personal well-being in terms of heat or dehydration.  Participants who had worn a hood responded as follows.  **Safety:** low or very low risk, 33 (92%); high risk, 2 (5%).  **Communication:** no or minor impairment, 17 (47%); some or major impairment, 19 (53%)  **Ability to provide care:** no or minor reduction, 26 (73%); important or major reduction, 10 (28%)  **Heat and dehydration:** no or minor issue, 14 (39%); significant or major issue, 24 (61%)  **Comfort:** comfortable or fairly comfortable, 23 (63%); uncomfortable or fairly uncomfortable, 13 (37%)  Some suggestions for improvement were made.   - Hood and respiratory protection could be designed in one piece. - The head cover could incorporate eye protection so that goggles are not necessary. - Light suits with powered air purifying respirator (PAPR) helmets would probably be more suitable for medical personnel. |
| Resource use | Hair cover, US$ 0.002  Hood for coverall, US$ 0.68 |
| Feasibility | Combining the hood with a face shield may be challenging. When the face shield is worn under the hood, the angle of the shield to the face may be increased, thus increasing the risk of splashes; however, when the face mask is worn over the hood, it has to be removed early in the undressing procedure, whereas it would be preferable to leave the face shield on until a later stage. Similar potential issues with regard to combining the hood with goggles, surgical or medical mask or particulate respirator were unclear. |
| Applicability | This recommendation is applicable to health workers involved in caring for patients suffering from filovirus disease. |
| Implementation considerations | - Specifications - Training - Waste disposal - Availability - WHO should develop specifications and training materials on use of PPE and disposal protocols |
| Research priorities | Observational studies on compliance, staff surveys on perceived comfort and barriers to compliance. |
