# Supplementary material for: Incorporating health workers’ perspectives into a WHO guideline on personal protective equipment developed during an Ebola virus disease outbreak
Source: F1000Res. 2018 Mar 9;7:45. Originally published 2018 Jan 11. [Version 2] doi: 10.12688/f1000research.12922.2 (PMC5820616; doi:10.12688/f1000research.12922.2)
Supplement: Peer review history

# Peer review: Referee response for version 1

**DOI:** 10.5256/f1000research.14011.r30694

**Published:** 2018-02-20

**Authors:** Elizabeth L. Beam

**Affiliations:** 1 College of Nursing, University of Nebraska Medical Center, Omaha, NE, USA

I had difficulty reading the tables in the article. I thought maybe it was the way they were displaying on my computer, but nothing seemed to change when I clicked on them. Please make these charts simple to read and clear. I need to see the tables to make sure your findings are adequately described.

 The article is really well written. I was very pleased with the quality of the writing and the honesty of the authors about their challenges. This is important work in the area of PPE use.

 While I know that this was quick work in a difficulty setting, I still feel like the article needs to do justice to personal protective equipment research of the past 20 years (at least since SARS). The major section that needs more referencing is the discussion section.  How do your findings compare to what we have found in epidemiological studies, simulation studies, and others on PPE. Even if these studies were not done in the context of an outbreak of EVD in Africa, they should still be discussed. There is literature on some of these areas that would bring worthwhile context to your findings.

I have read this submission. I believe that I have an appropriate level of expertise to confirm that it is of an acceptable scientific standard.

# Response

**Published:** 2018-03-05

**Authors:** Saskia Den Boon

**Affiliations:** World Health Organization, Switzerland

I had difficulty reading the tables in the article. I thought maybe it was the way they were displaying on my computer, but nothing seemed to change when I clicked on them. Please make these charts simple to read and clear. I need to see the tables to make sure your findings are adequately described.

Author’s response: Thank you for reviewing and approving our paper. We assume that you are referring to table 2. We have revised the table by adding the categories that were previously omitted from the table. For example, in version 1 we only presented in the table the number health workers that indicated they felt at extremely low or low risk, but in version 2 we have added a column indicating the number of health workers feeling at high or extremely high risk. We have also added a foot note explaining how the denominator in each cell reflects missing values for that particular question. We hope that this has improved the readability of the tables.

 The article is really well written. I was very pleased with the quality of the writing and the honesty of the authors about their challenges. This is important work in the area of PPE use.

Author’s response: Thanks for these kind words about our study.

 While I know that this was quick work in a difficulty setting, I still feel like the article needs to do justice to personal protective equipment research of the past 20 years (at least since SARS). The major section that needs more referencing is the discussion section.  How do your findings compare to what we have found in epidemiological studies, simulation studies, and others on PPE. Even if these studies were not done in the context of an outbreak of EVD in Africa, they should still be discussed. There is literature on some of these areas that would bring worthwhile context to your findings.

Author’s response: Thanks for this suggestion. We have added a number of references to the literature to our discussion section.

# Peer review: Referee response for version 1

**DOI:** 10.5256/f1000research.14011.r30348

**Published:** 2018-01-29

**Authors:** Patrick Mbah Okwen, Miriam N. Nkangu

**Affiliations:** 1 Bali and Centre for Development of Best practices in Health Yaounde (CDBPH), Bali District Hospital, Yaoundé, Cameroon; 2 School of Epidemiology and Public Health, University of Ottawa, Ottawa, ON, Canada

Well written paper on an important and largely ignored subject: ‘health workers perspectives for guidelines’; Also on top global health issue ‘Ebola virus disease’. Study process was speedy and appropriate for the urgency needed for guidelines to be developed making this a good learning experience. However, there are a few points of attention listed below. I have also highlighted the sections relevant to my comments here.

**Methods**

**AQ1:**

 ‘The 2014–2016 EVD outbreak in West Africa was initially declared a Public Health Emergency of International Concern in early August 2014, coinciding with the decision to develop a WHO rapid advice guideline on the selection and use of PPE for EVD care in outbreaks.’

 This statement will fit more within the background section, consider moving into background.

**AQ2:**

 ‘We electronically surveyed international frontline physicians and nurses who participated in foreign medical teams deployed to the affected countries in early stages of the EVD outbreak.’

 Clearly stating time frame in the methods section within which survey was done will also be helpful for readers, although a time frame is given later under participants, it is not clear if this was for survey or the sampling. this time frame is also very early in the outbreak

**AQ3:**

 Settings is not well described, consider discussing setting in more detail under a separate title.

**Results**

**AQ4:**

 Clinicians express discomfort and safety, it may be interesting to know if at some point in the interviews they weighed in on safety versus comfort e.g. will the feeling of safety make them cope with discomfort? Or does discomfort make safety inconsequential?

 I have answered ‘Partly’ to the question “Is the work clearly and accurately presented and does it cite the current literature?” as a small part of the methods may benefit clarity if texts are moved around.

 I have answered ‘Partly’ to the question “Are the conclusions drawn adequately supported by the results?” as it will be important to discuss discomfort versus safety of risk or clearly state if this was not evaluated by the study.

**Miriam N. Nkangu comments**

 This is an interesting piece and important in the context of infectious diseases. I will like to appreciate the authors for taking the initiative during such an emergency to collect such data. I will recommend the paper to be considered for indexing especially as it contributes towards developing guidelines for PPE which was more of a challenge to health workers during the outbreak. Understanding their challenges and experiences especially in very humid temperatures is important. Most importantly, the 2014 outbreak was a remarkable and most catastrophic outbreak. Thus, using the outbreak as a point of focus adds value to the work considering that it pulled health workers from various countries.

**Methods, Background/limitation**

- Why only physicians and nurses perspectives regarding PPE? I understand the relative risk for physicians and nurses as frontline workers is high, but other health workers are involved, and have recorded fatality rates, their experiences with PPE may also add value especially in the context of developing guidelines. Maybe the authors should consider adding this to limitations.

**Methods**

- Four or five Likert is not explicit; it does not tell which questions were measured using scales of four and which used five and how they way categorize for-example., 1 indicating low or high? Agree or somewhat agree?
- Understand the sample size was small and is actually mentioned as a limitation, however, any data on number of nurses and physicians that were deployed by WHO and MSF during the period of data collection for background purposes and to justify the limitation?
- The sentence under data analysis is not clear to me, maybe rephrasing to better explain to the audience “For the purpose of statistical analysis, we considered each participant’s experience with a PPE item unique and independent.”
- The survey assumes that all the participants speak and write English? Language characteristic not mentioned considering that these affected countries some are French countries. If all participants were not English speaking how was it translated? Especially as the authors mentioned that respondents could not comprehend some questions due to time constraint.

 

 The literature highlights some gender differences for PPE amongst physicians and nurses especially in African context-assuming nurses are mostly women and physicians men- -it would have been good to explore differences between nurses and physicians with regards to the specific PPE used. Were physicians exposed to more sophisticated PPE than nurses?

 Other comments that may be of interest to the authors:

 I understand the limitation of the paper is focused on participants in Ebola treatment centers and only foreign deployed. However, guidelines should take into consideration local reality in terms of culture? Based on previous outbreaks, most families prefer to care for patient at home and given the limited resources in this context; Local materials were used at home in 2014 as PPE http://www.cnn.com/2014/09/25/health/ebola-Fatu-family/index.html.

 Given the reality of limited resources, and the fact that most families prefer to care for patient at home it would add more value also to consider experiences of those who cared for patient at home, the type of PPE used and opportunities in incorporating local reality into evidence-based guidelines for PPE.

We have read this submission. We believe that we have an appropriate level of expertise to confirm that it is of an acceptable scientific standard.

# Response

**Published:** 2018-03-05

**Authors:** Saskia Den Boon

**Affiliations:** World Health Organization, Switzerland

Well written paper on an important and largely ignored subject: ‘health workers perspectives for guidelines’; Also on top global health issue ‘Ebola virus disease’. Study process was speedy and appropriate for the urgency needed for guidelines to be developed making this a good learning experience. However, there are a few points of attention listed below. I have also highlighted the sections relevant to my comments here.

Author’s response: Thank you for reviewing and approving our paper. See below our responses to your comments. We have made a number of changes in the text in response to your comments.

**Methods**

**AQ1:**

 ‘The 2014–2016 EVD outbreak in West Africa was initially declared a Public Health Emergency of International Concern in early August 2014, coinciding with the decision to develop a WHO rapid advice guideline on the selection and use of PPE for EVD care in outbreaks.’

 This statement will fit more within the background section, consider moving into background.

Author’s response: We removed this sentence from the methods section and have added it, slightly modified, to the background section.

**AQ2:**

 ‘We electronically surveyed international frontline physicians and nurses who participated in foreign medical teams deployed to the affected countries in early stages of the EVD outbreak.’

 Clearly stating time frame in the methods section within which survey was done will also be helpful for readers, although a time frame is given later under participants, it is not clear if this was for survey or the sampling. this time frame is also very early in the outbreak

Author’s response: We send out the request for participation in September 2014 and have added this to the methods section. Later we indicate that the survey was open for a 10-day period. The health workers eligible for participation were those who were deployed to West Africa between March and September 2014 which was already stated in the methods section under participants.

**AQ3:**

 Settings is not well described, consider discussing setting in more detail under a separate title.

Author’s response: We are not sure how to respond to this question of the reviewer. We did an online survey among health workers who were deployed by MSF or WHO to respond to the Ebola outbreak in West Africa early on in the epidemic. Health workers worked in local hospitals, clinics or Ebola Treatment Centers, but because we did not ask further information about these settings we cannot provide a more detailed description.

**Results**

**AQ4:**

 Clinicians express discomfort and safety, it may be interesting to know if at some point in the interviews they weighed in on safety versus comfort e.g. will the feeling of safety make them cope with discomfort? Or does discomfort make safety inconsequential?

Author’s response: We assume that the reviewer is referring to question 11 which asked, “please indicate how safe you felt by ticking a box for each aspect of Personal Protective Equipment”. As we have stated in the discussion, survey participants had difficulty answering this question because of the way the answer categories were phrased, e.g. “extremely low risk, I felt comfortable”. In this answer category we wanted comfortable to mean “I am not worried about safety”, but this was sometimes interpreted as “I am physically comfortable (e.g. not overheated, etc.)”. If we had had more time for piloting, we would have been able to pick this up before sending out the survey. However, through comments from health workers it became clear that they indeed cope with discomfort because the PPE makes them feel safe and we have added the following sentence to the discussion: “Health workers accept a certain degree of discomfort in return for the protection provided by PPE”.

 I have answered ‘Partly’ to the question “Is the work clearly and accurately presented and does it cite the current literature?” as a small part of the methods may benefit clarity if texts are moved around.

Author’s response: we hope that our amendments have improved the methods section.

 I have answered ‘Partly’ to the question “Are the conclusions drawn adequately supported by the results?” as it will be important to discuss discomfort versus safety of risk or clearly state if this was not evaluated by the study.

Author’s response: we hope that our amendment has taken away the concern of the reviewer.

**Miriam N. Nkangu comments**

 This is an interesting piece and important in the context of infectious diseases. I will like to appreciate the authors for taking the initiative during such an emergency to collect such data. I will recommend the paper to be considered for indexing especially as it contributes towards developing guidelines for PPE which was more of a challenge to health workers during the outbreak. Understanding their challenges and experiences especially in very humid temperatures is important. Most importantly, the 2014 outbreak was a remarkable and most catastrophic outbreak. Thus, using the outbreak as a point of focus adds value to the work considering that it pulled health workers from various countries.

Author’s response: Thank you for reviewing our paper and for making helpful comments and suggestions. See below our responses.

**Methods, Background/limitation**

- Why only physicians and nurses perspectives regarding PPE? I understand the relative risk for physicians and nurses as frontline workers is high, but other health workers are involved, and have recorded fatality rates, their experiences with PPE may also add value especially in the context of developing guidelines. Maybe the authors should consider adding this to limitations.

Author’s response: We agree with the reviewer about the importance of PPE for other health workers, for example cleaners, laboratory workers, burial teams and other workers. However, the focus of the WHO guideline which our study aimed to inform, was on healthcare workers and therefore we also focused our survey on this group.

**Methods**

- Four or five Likert is not explicit; it does not tell which questions were measured using scales of four and which used five and how they way categorize for-example., 1 indicating low or high? Agree or somewhat agree?

Author’s response: We agree with the reviewer that it would have been better to have used a comparable (e.g. 5-point scale) for all the questions. If we had more time for piloting, we may have picked this up before sending out the survey. Now, the questions on safety and comfort had a 4-point scale and questions on communication, ability to provide care, and heat and dehydration had a 5-point scale. As can be seen in the questionnaire which is included in the supplementary material, we did not use coding in the answer categories. 

- Understand the sample size was small and is actually mentioned as a limitation, however, any data on number of nurses and physicians that were deployed by WHO and MSF during the period of data collection for background purposes and to justify the limitation?

Author’s response: As stated in the results section, we invited 192 health workers (166 from MSF and 26 from WHO) to participate in the survey, but this included health workers outside the sampling frame (e.g. logisticians and water, sanitation and hygiene experts). Unfortunately we do not have more detailed information on numbers deployed.  

- The sentence under data analysis is not clear to me, maybe rephrasing to better explain to the audience “For the purpose of statistical analysis, we considered each participant’s experience with a PPE item unique and independent.”

Author’s response:  We have now added the following clarification to the methods section: “i.e.  we did not account for the fact that the experience came from one and the same health worker”.

- The survey assumes that all the participants speak and write English? Language characteristic not mentioned considering that these affected countries some are French countries. If all participants were not English speaking how was it translated? Especially as the authors mentioned that respondents could not comprehend some questions due to time constraint.

Author’s response:  Yes, this is correct. We assumed that all participants could speak and write English and we did not translate the questionnaire. The miscomprehension was due to the fact that two questions were not phrased clearly, rather than the language skills of the survey participants.

 The literature highlights some gender differences for PPE amongst physicians and nurses especially in African context-assuming nurses are mostly women and physicians men- -it would have been good to explore differences between nurses and physicians with regards to the specific PPE used. Were physicians exposed to more sophisticated PPE than nurses?

Author’s response:  This is a very interesting question. Although our study was not designed to answer this question and the number of participants was too small to do any stratified analysis, I had a brief look at the data. We indeed found a higher proportion of physicians among males (91%) than among females (57%), but there were no obvious differences in robustness of PPE, when I compared gown or coverall use between males and females, or between physicians and nurses (varying between 32-36% using a gown).

 Other comments that may be of interest to the authors:

 I understand the limitation of the paper is focused on participants in Ebola treatment centers and only foreign deployed. However, guidelines should take into consideration local reality in terms of culture? Based on previous outbreaks, most families prefer to care for patient at home and given the limited resources in this context; Local materials were used at home in 2014 as PPE http://www.cnn.com/2014/09/25/health/ebola-Fatu-family/index.html.

 Given the reality of limited resources, and the fact that most families prefer to care for patient at home it would add more value also to consider experiences of those who cared for patient at home, the type of PPE used and opportunities in incorporating local reality into evidence-based guidelines for PPE.

Author’s response:  We acknowledge the importance of this issue brought up by the reviewer but it fell outside the scope of the study and the WHO guideline that we were aiming to inform.
